# Natural Healing

**Published:** 1886-05

**Authors:** 


### NATURAL HEALING.

New York, April 19th, 1886.

SIR:—

I have been a constant reader of "Hall's Journal of Health" for a number of years, and at this late day I am astonished to find such an article as the April number contains, entitled "Natural Healing," admitted to its columns. What does it mean? Is this time honored monthly to become the organ of a class of pretenders, ignorant alike of the simplest rules of surgery and therapeutics? If so, it is high time that such of your readers as have devoted years to the study of medical science should be apprised of it, that they may hold an equivocal attitude towards a "publication" which they have been want to regard as friendly to this object.

M. D.

It is seldom that we take any notice of anonymous communications, but the foregoing has something of a representative character, if we may judge from the signature. The writer is doubtless a devotee of the old school of medicine, which has held its way in colleges, in legislatures, in families, and we may say in churches and *cemeteries* time immemorial, and only reformed its mistakes when driven to it by outside pressure. We are free to say that we are not wedded to any special system or school, and mean to give our patrons well attested facts of cure, by any and all known methods. If M. D. shall for this, discard the Journal, we must yield gracefully to the withdrawal of his patronage as a *reader*, always regretting our inability to kindle in him a desire to learn anything new under the sun. The renowned "Harvey" found this a most unprofitable endeavor with men of his class, who take their modicum of knowledge, as *bon vivants* take their wine, out of long stored bottles, the older and cobwebier the better. The first number of the present volume clearly foreshadowed what our future course would be, and we mean to make our promises good to our readers at large. In proof of this we offer the contents of the present number.

THE MANAGER.

**Phthisis a Modern Disease.**—Dr. Hunt, of Sheffield, argues that phthisis is a modern disease, and points out that the countries most free from it are the northern and colder regions. He recommends the selection of such countries for the cure of pythisical patients. He expresses himself as very skeptical of all medicines as remedical agents, having no faith in antiseptic inhalations, iodoform, etc. He is of opinion that, in the future, the treatment will come more under the domain of surgery, and that the drainage of a lung cavity will be as recognized an operation as the drainage of an abscess of the hip.

